# Chewable tablets containing rape bee pollen and maca attenuate testosterone propionate–induced benign prostatic hyperplasia in Sprague–Dawley rats by regulating the gut microbiota and modulating the IL-6/JAK2/STAT3 signaling pathway

**DOI:** 10.3389/fmicb.2025.1547724

**Published:** 2025-11-18

**Authors:** Xiaoqiang Huang, Guohua Ru, Hengyun Tian, Pengbo Zhang, Hongchang Zhao, Shouren Zhang, Sijie Wang, An Jia, Wanli Zhao

**Affiliations:** 1 School of Medicine, Henan Provincial Key Laboratory of Nanocomposites and Applications, Institute of Nano-Structured Functional Materials, Huanghe Science and Technology University, Zhengzhou, China; 2 School of Pharmacy, Henan University, Kaifeng, China; 3 Jiangsu Key Laboratory for the Research and Utilization of Plant Resources, Institute of Botany, Jiangsu Province and Chinese Academy of Sciences (Nanjing Botanical Garden, Mem. Sun Yat-Sen), Nanjing, China

**Keywords:** benign prostatic hyperplasia, rape bee pollen, maca, gut microbiota, IL-6/JAK2/STAT3 signaling pathway

## Abstract

**Background:**

Benign prostatic hyperplasia (BPH) significantly compromises patients' quality of life, has detrimental effects on both physical and mental well-being, and poses a substantial economic burden. Current pharmacological interventions primarily focus on symptom relief; nevertheless, their associated adverse effects are evident. Rape bee pollen and maca are both homologous food products, which are not only rich in nutrition, but also have many biological activities. There is no report on the combination of rape bee pollen and maca in benign prostatic hyperplasia therapy, and no report on the molecular mechanism. This study aims to systematically investigate the therapeutic efficacy and underlying molecular mechanisms of a chewable tablet formulation containing rape bee pollen and maca in the management of BPH.

**Methods:**

First, the chewable tablets were prepared from rape bee pollen and maca, and the High Performance Liquid Chromatography (HPLC) fingerprint was established. During a 4-week period, rats were subjected to subcutaneous injections of testosterone propionate solution to establish a BPH model, while simultaneously receiving orally administered chewable tablets for BPH treatment via gavage. We assessed the body weight, prostate tissue index, serum levels of dihydrotestosterone (DHT), testosterone (T), estradiol (E2), interleukin-6 (IL-6), tumor necrosis factor-alpha (TNF-α), interleukin-1beta (IL-1β), superoxide dismutase (SOD), malondialdehyde (MDA) and glutathione (GSH) levels in rats. Prostate tissues were collected for histopathological analysis. The protein expression levels of IL-6, phosphorylated Janus kinase 2 (*p*-JAK2), and phosphorylated signal transducer and activator of transcription 3 (*p*-STAT3) were determined by Western blotting. Rat feces were collected aseptically for 16S rDNA gene sequencing to assess gut microbiota levels. Additionally, our research findings demonstrate that the combined application of rape pollen and maca exhibits superior therapeutic efficacy compared to the individual use of either rape pollen or maca alone.

**Results:**

We prepared the rape bee pollen and maca chewable tablets, established the HPLC fingerprint map, and quantitatively analyzed four of these main components. We found that the rape bee pollen and maca chewable tablets significantly inhibited DHT, T, E2, E2/T ratio, IL-6, TNF-α, and IL-1β and reduced MDA levels and increased SOD, GSH levels. 16S rDNA gene sequencing revealed that the chewable tablet increased the abundance of the beneficial bacteria *Faecalibacterium, Collinsella, Phascolarctobacterium, Kineothrix*, and *Lactobacillus*, and decreased that of the pro-inflammatory *bacterium Prevotella*. Western blot indicated that the chewable tablet could significantly inhibit the IL-6, *p*-JAK2, and *p*-STAT3 protein expression levels.

**Conclusion:**

We found that rape bee pollen and maca chewable tablets had a protective effect on rats with BPH, and also found that the combination of rape bee pollen and maca was better than separate application. The mechanism may be related to the gut microbiota and IL-6/JAK2/STAT3 signaling pathway.

## 1 Introduction

Benign prostatic hyperplasia (BPH) is a highly prevalent condition in elderly males, with approximately 30%−40% of men affected by age 40 and 70%−80% of men affected by age 85 (Cannarella et al., 2021; Welén and Damber, 2022). This disorder leads to lower urinary tract symptoms (LUTS), including urinary hesitation, intermittent urinary stream, and straining during urination, thereby significantly compromising patients' quality of life and imposing substantial physical, mental, and economic burdens globally (Ho and Habib, 2011; Song et al., 2023). Recent global statistics indicate that over 94 million men worldwide experience BPH-related symptoms as of 2019 (Awedew et al., 2022). The pathophysiology of BPH involves imbalances in epithelial and stromal cell proliferation and apoptosis within the prostate's transition zone, leading to glandular and stromal hyperplasia (Shah et al., 2021). Specifically, epithelial hyperplasia forms larger glandular nodules, while stromal hyperplasia contributes to diffuse extracellular matrix deposition, particularly of type 1 collagen (Hata et al., 2023). These structural changes collectively disrupt urinary function and exacerbate LUTS severity, underscoring the need for effective therapeutic interventions.

Disease development and treatment are closely intertwined with the gut microbiome. Accumulating evidence has established a bidirectional relationship between BPH and gut microbiota dysregulation. For instance, recent studies elucidating the gut–prostate axis have revolutionized our understanding of BPH pathogenesis (Liu et al., 2021; Li et al., 2024). Animal models further revealed that high-fat diet-induced imbalances in the gut microbiome—particularly elevated ratios of *Bacteroidetes* to *Firmicutes*—are associated with BPH development (Gu et al., 2021). Therapeutic interventions such as finasteride treatment restored the abundance of beneficial gut bacteria, including *Lactobacillus* and *Acetatifactor*, to baseline levels in BPH patients (An et al., 2023). Mechanistically, gut microbiota perturbations may disrupt prostate homeostasis through immune activation and proinflammatory cytokine secretion, thereby promoting pathological inflammation (Russo et al., 2023).

Chronic inflammation has been identified as a key factor in the development and course of certain human illnesses, including prostate cancer and BPH. Interleukin-6 (IL-6) is a cytokine from the gp130 family that mediates the process of inflammation (Staršíchová et al., 2010). The IL-6/JAK2/STAT3 pathway is a key inflammatory signaling pathway. Numerous physiological and pathological processes, including angiogenesis, immunological control, and cell division and proliferation, are influenced by this pathway (Huang et al., 2022). Excessive IL-6 production in response to inflammatory stimulation is an effective stimulator of the JAK/STAT signaling pathway. IL-6 may contribute to inflammation by activating this pathway, which promotes epithelial–mesenchymal transition (Xiao et al., 2017; Singh et al., 2020). Studies have demonstrated that canagliflozin delays the progression of BPH by inhibiting the JAK2/STAT3 pathway (Elbaz et al., 2023).

The primary pharmacological interventions for BPH remain 5α-reductase inhibitors, α1-receptor antagonists, and PDE5 inhibitors (Antoniou et al., 2023). Adverse events associated with 5α-reductase inhibitors encompass erectile dysfunction, ejaculatory disorders, mood disturbances (depression/anxiety), and an elevated risk of prostate cancer progression (Miernik and Gratzke, 2020; Stewart and Lephart, 2023). Meanwhile, α1-receptor blockers are characterized by side effects including orthostatic hypotension, sedation, ejaculatory abnormalities, cognitive impairment, and depressive symptoms. These limitations have catalyzed research interest in complementary/alternative therapies, particularly phytotherapeutic agents (Stewart and Lephart, 2023). Rape bee pollen (Brassica napus L.) emerges as a dual-use agent in traditional Chinese medicine and functional nutrition. Its bioactive profile comprises polyunsaturated fatty acids, flavonoids, and a comprehensive array of vitamins (Chen et al., 2020; Han et al., 2023), manifesting pleiotropic physiological activities including antioxidative, anti-inflammatory, immunomodulatory, antimicrobial, and organoprotective effects (Rodríguez-Pólit et al., 2023). Specifically, its extract modulates gut microbiota composition in BPH rat models by promoting beneficial taxa (*Coprococcus* and *Jeotgalicoccus*) while suppressing pathogenic microorganisms (*Turicibacter* and *Clostridiaceae_clostridium*) (Qiao et al., 2023). Additionally, rape bee pollen exerts anti-BPH activity through cyclooxygenase-2 (COX-2 pathway inhibition) (Yang et al., 2014). Maca, also known as Peruvian ginseng, was classified as a health food in 2011. Maca root contains numerous bioactive compounds, including macaamide, macaene, glucosinolates, polyphenols, and alkaloids, which can mitigate sexual dysfunction. It also has neuroprotective, memory-enhancing, antidepressant, antioxidant, anti–prostate hyperplasia, anti-inflammatory, and skin-protective properties (Da Silva Leitão Peres et al., 2020). Previous studies have demonstrated that maca can be utilized to treat BPH. Maca can decrease testosterone propionate–induced BPH by preventing dihydrotestosterone conversion (Zou et al., 2017) and can regulate inflammation in patients with BPH by boosting the levels of anti-inflammatory cytokines (IL-4, IFN-γ) and reducing those of pro-inflammatory cytokines (tumor necrosis factor-α) (Vásquez-Velásquez et al., 2020; Ulloa Del Carpio et al., 2024).

In summary, both rape bee pollen and maca exhibit therapeutic potential against BPH. This study formulated chewable tablets using these two ingredients to evaluate their synergistic effects in treating BPH, elucidate the underlying molecular mechanisms, and establish a standardized fingerprint chromatogram for quality control purposes.

## 2 Materials and methods

### 2.1 Chemicals and reagents

Testosterone propionate was purchased from Aladdin (Shanghai, China), maca was purchased from Beijing Tongrentang Co., Ltd (Beijing, China), and rape bee pollen was purchased from Henan Mileyuan Beekeeping Specialized Cooperative (Henan, China). Lactose, microcrystalline cellulose and magnesium stearate were purchased from Shaanxi Zhengyi Pharmaceutical Auxiliary Materials Co., Ltd. (Shanxi, China). Citric acid was purchased from Henan Wanbang Chemical Co., Ltd. (Henan, China) and finasteride from HangZhou MSD Pharmaceutical Co., Ltd (Hangzhou China). ELISA kits for the determination of IL-6, TNF-α, and IL-1β in rats were acquired from MultiSciences Lianke Biotech Co., Ltd. (Hangzhou, China). Anti-JAK2, anti–IL-6, anti–phospho-STAT3, anti-STAT3, and anti–phospho-JAK2 were purchased from Chengdu Zhengneng Biotechnology Co., Ltd. (Chengdu, China); Standard products such as rutin, quercetin, kaempferol, Isorhamnetin, etc. were purchased from Shanghai Yuanye Biotechnology Co., Ltd. (Shanghai, China); chromatography-grade phosphoric acid (Tianjin Comio Chemical Reagent Co., Ltd.).

### 2.2 Chewable tablet preparation

The chewable tablets are prepared using a wet granulation process, with maca water extract and rape bee pollen as principal ingredients, and lactose, microcrystalline cellulose, citric acid, and magnesium stearate as auxiliary components. Each chewable tablet weighs 450 mg and contains the following ingredients: rape bee pollen 136.62 mg, maca 136.62 mg, microcrystalline cellulose 85.545 mg, lactose 83.745 mg, citric acid 2.97 mg, and magnesium stearate 4.5 mg.

### 2.3 Preparation of the standard solution and sample solution

Take the appropriate amount of each control product, add methanol to make the mixed control solution of rutin, quercetin, kaempferol and Isorhamnetin quality concentration of 12.74 μg/mL, 34.12 μg/mL, 76.56 μg/mL, and 10.23 μg/mL, respectively. Shake well and set aside.

The 10 batches of rape bee pollen maca masticable tablets were prepared according to the “2.2” method. Each sample was weighed 1.0 g, placed in 100 mL round bottom flask, add 50 mL of methanol and 10 mL of 25% hydrochloric acid solution, weighed, heat reflux for 60 min, cool, weigh the mass with methanol, shake well, filter 0.22 μm microporous filter membrane, take the continued filtrate, and set aside.

### 2.4 Instruments and chromatographic conditions

Chromatographic conditions: Chromatographic column: Agilent ZORBAX SB-C18 column (4.6 × 250 mm, 5 μm), mobile phase: methanol (A)-0.4% phosphoric acid water (B), gradient elution: 0–30 min (A: 30%−50%), 30–50 min (A: 50%−55%), 50–51 min (A: 55%−30%), 51–60 min (A: 30%−30%), detection wavelength: 368 nm, The flow rate was 1 mL/min, the injection volume was 10 μL, and the column temperature was 30°C.

### 2.5 Animals experiment

After a week-long acclimation period, male Sprague—Dawley rats were randomized into 8 groups with 10 rats per group using a weight-based stratified random sampling technique, which guaranteed that the average weight of rats in each group was the same. Rats were divided into the following 8 groups: control group, model group, finasteride group (0.52 mg/kg), rape bee pollen group (630.0 mg/kg), maca group (630.0 mg/kg), and Chewable Tablets at 3 different dosages groups, namely, low (518.7 mg/kg), middle (1,037.5 mg/kg), and high (2,075.0 mg/kg) dosages. BPH was induced in rats in the model and drug-administered groups using a subcutaneous injection of testosterone propionate (5 mg/kg/day dissolved in olive oil) for 28 consecutive days. Only olive oil was administered subcutaneously for 28 days to rats in the control group. Along with the testosterone propionate injection, rats in the drug-administered groups received daily gavage for 28 days, whereas those in the control group received distilled water following the same protocol. Fecal samples were obtained from each group of rats on the day before the experiment ended. After overnight fasting, blood was drawn from the abdominal aorta under anesthesia with 2% sodium pentobarbital (50 mg). The blood samples were centrifuged at 4,000 rpm for 15 min to obtain the serum. After prostate tissues were imaged for morphological evaluation, they were quickly separated and weighed. The serum was collected and stored at −80°C for ELISA. Rat feces were stored at −80°C until they were used for gut microbiome identification. A part of the prostate tissues was fixed in 4% paraformaldehyde for further histological evaluation. The remaining prostate tissues were promptly stored at −80°C for western blotting.

### 2.6 Oxidative stress

Serum superoxide dismutase (SOD), malondialdehyde (MDA) and glutathione (GSH) levels were measured using commercial reagents (Nanjing Jiancheng Bioengineering Institute, Nanjing, China) according to the manufacturer's recommendations.

### 2.7 Histopathological analysis

H&E staining was used to determine the morphological characteristics of rat prostate glands that were obtained after treatment with testosterone propionate. The epithelial thickness was determined using ImageJ software.

### 2.8 Enzyme-linked immunosorbent assay

Three types of pro-inflammatory cytokines, i.e., IL-1β, IL-6, and TNF-α, were measured in serum supernatant using the rat ELISA Commercial Kit (MultiSciences Lianke Biotech, Hangzhou, China) according to the manufacturer's recommendations.

### 2.9 Gut microbiota analysis

The feces of rats in the control group, model group, chewable tablets middle dosages group, and finasteride group were used as test samples. The gut microbiome samples were sent to BGI Co., Ltd, China (Shenzhen, China) for DNA extraction and sequencing of 16S rRNA gene. Total genomic DNA of the gut microbiome was extracted using E.Z.N.A.^®^ Bacterial DNA Kit (Omega Bio-tek, Norcross, GA, USA) according to manufacturer's instruction. The V4 of 16S rRNA gene from the single gut microbiome sample was amplified. After genome DNA was normalized to 30 ng per PCR reaction, V4 dual-index fusion PCR primer cocktail and PCR master mix were added, then to run PCR. The melting temperature was 56°C and PCR cycle was 30. The PCR products were purified with AmpureXP beads to remove the unspecific products. The resulting library was used for sequencing on Illumina HiSeq 2500 platform following the standard pipelines of Illumina, and generating 2 × 250 bp paired-end reads (Sinclair et al., 2015).

To obtain clean reads, the raw data were filtered to eliminate the adapter pollution and low-quality reads by an in-house procedure as following (Fadrosh et al., 2014): The clean paired-end reads with overlap were merged to tags using FLASH (fast length adjustment of short reads, v1.2.11) (Magoč and Salzberg, 2011). Then, the tags were clustered to OTUs at 97% sequence similarity by scripts of software USEARCH (v9.1.13) (Edgar, 2013). Taxonomic ranks were assigned to OTUs representative sequence using Greengene (v201305, https://docs.qiime2.org/2024.2/data-resources/#greengenes) (Cole et al., 2014). At last, alpha diversity, beta diversity and the different species screening were analyzed based on OTUs and taxonomic ranks using mother (v1.31.2), software R (v3.1.1), QIIME (v1.80), or metastats (https://huttenhower.sph.harvard.edu/metastats/) (Schloss et al., 2009; White et al., 2009).

### 2.10 Western blot analysis

The total protein was isolated from prostate tissue homogenate by Radioimmunoprecipitation assay buffer. The protein concentration was then determined using the BCA assay kit (Nanjing Jiancheng Bioengineering Institute, Nanjing, China). Protein samples were separated on a 10% SDS-PAGE gel and transferred to a 0.45 μm polyvinylidene fluoride (PVDF) membrane, and then the PVDF membrane was blocked with 5% skim milk or 5% bovine serum albumin for 2 h. The blocked PVDF membrane were incubated with the following primary antibodies; p-JAK2 (1:1000), JAK2 (1:1000), p-STAT3 (1:1000), STAT3 (1:1000), β-Actin (1:5000) diluted with 5% bovine serum albumin overnight at 4°C, The PVDF membrane was washed 3 times with 1 × TBST on a shaker, 10 min each time. The membrane was then incubated with HRP-labeled secondary antibodies for 2 h at room temperature, and the immunobands were visualized using chemiluminescent reagents in the Chemi-Doc XRS system, and the gray value of the protein bands was analyzed by Image J software. All the antibodies listed in this section were obtained from Chengdu Zhengneng Biotechnology Co., Ltd. (Chengdu, China).

### 2.11 Statistical analysis

Data computations and graphical representations were executed using GraphPad Prism software (version 9.5; San Diego, CA, United States). Intergroup disparities were examined using independent sample t-test, one-way analysis of variance, and Kruskal—Wallis test. Correlation was determined using the Spearman rank correlation coefficient (with a significance threshold of p < 0.05) in the corrplot software package of R (v3.4.1). GraphPad Prism v9.5 and R were used for analyses and figure generation. p < 0.05 was considered to be statistically significant. The results were expressed as mean ± standard deviations.

## 3 Results and discussion

### 3.1 HPLC fingerprint

**Figure 1:**
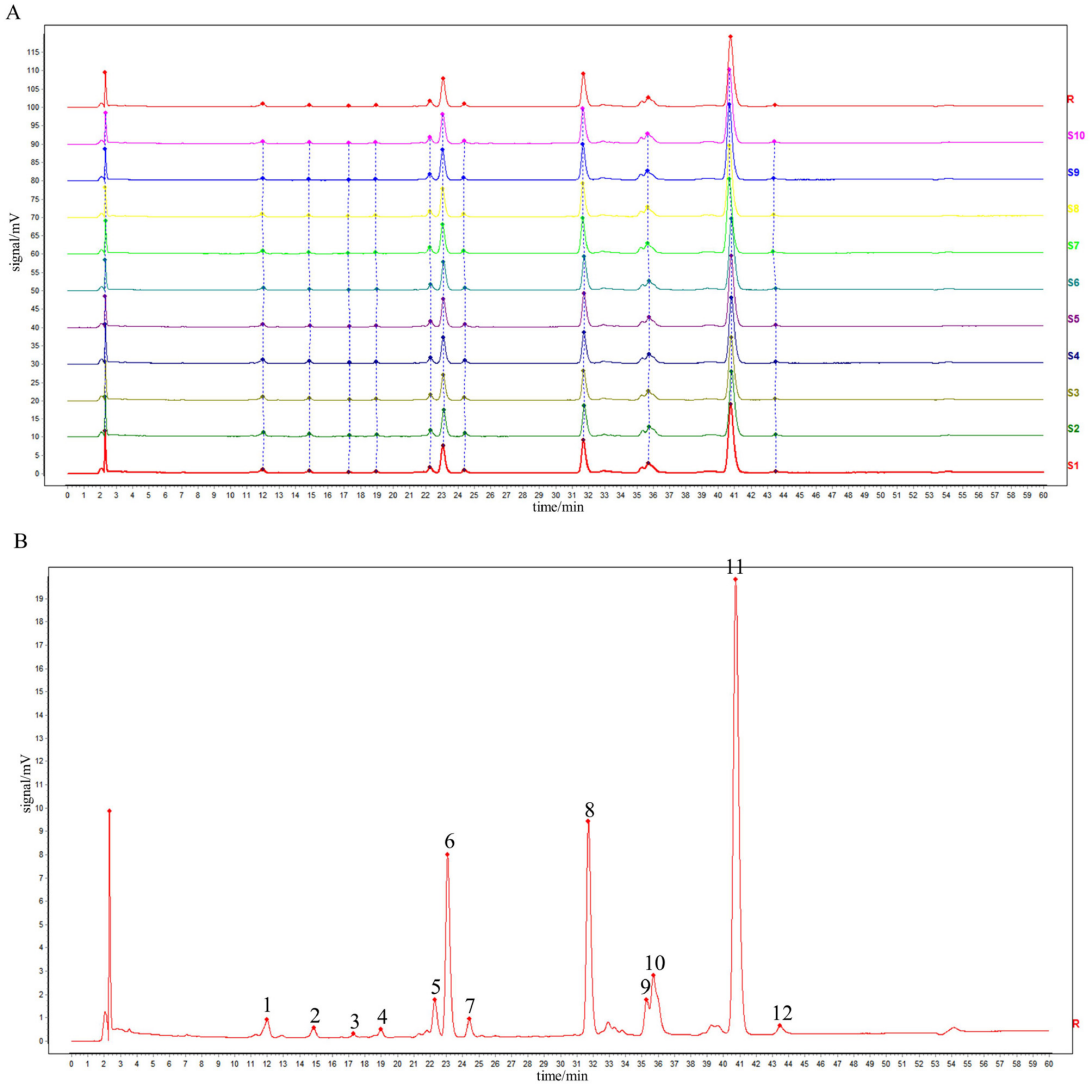
HPLC fingerprints of 10 batches of sample **(A)** and reference fingerprint chromatogram **(B)**.

Prepare 10 batches of rape bee pollen maca chewable tablet test solution (numbered S1~S10), measure according to the chromatographic conditions under item “2.4”, and record the chromatogram for 60 min. “Chinese traditional medicine chromatographic fingerprint similarity evaluation system (2012, 1 Edition)” was used to evaluate 10 batches of AIA data of sample to be tested. The chromatographic fingerprint of sample S1 was designated as the reference standard. Following multi-point calibrated full-spectrum peak alignment, a consensus fingerprint was generated using the median method, resulting in the identification of 12 characteristic common peaks. Among these, four peaks were structurally authenticated: Peak 4 (rutin), Peak 8 (quercetin), Peak 11 (kaempferol), and Peak 12 (isorhamnetin). Similarity analysis between the reference fingerprint and those of 10 batches of rape pollen-maca chewable tablets demonstrated high consistency, with similarity indices ranging from 0.999 to 1.000. As shown in Figures 1A, B and Table 1.

**Table 1:** Similarity results for the 10-batch samples.

**Sample number**	**Similarity results**	**Sample number**	**Similarity results**
S1	1.000	S6	0.999
S2	0.999	S7	0.999
S3	1.000	S8	1.000
S4	1.000	S9	1.000
S5	1.000	S10	1.000

### 3.2 The content of four components in the sample

**Figure 2:**
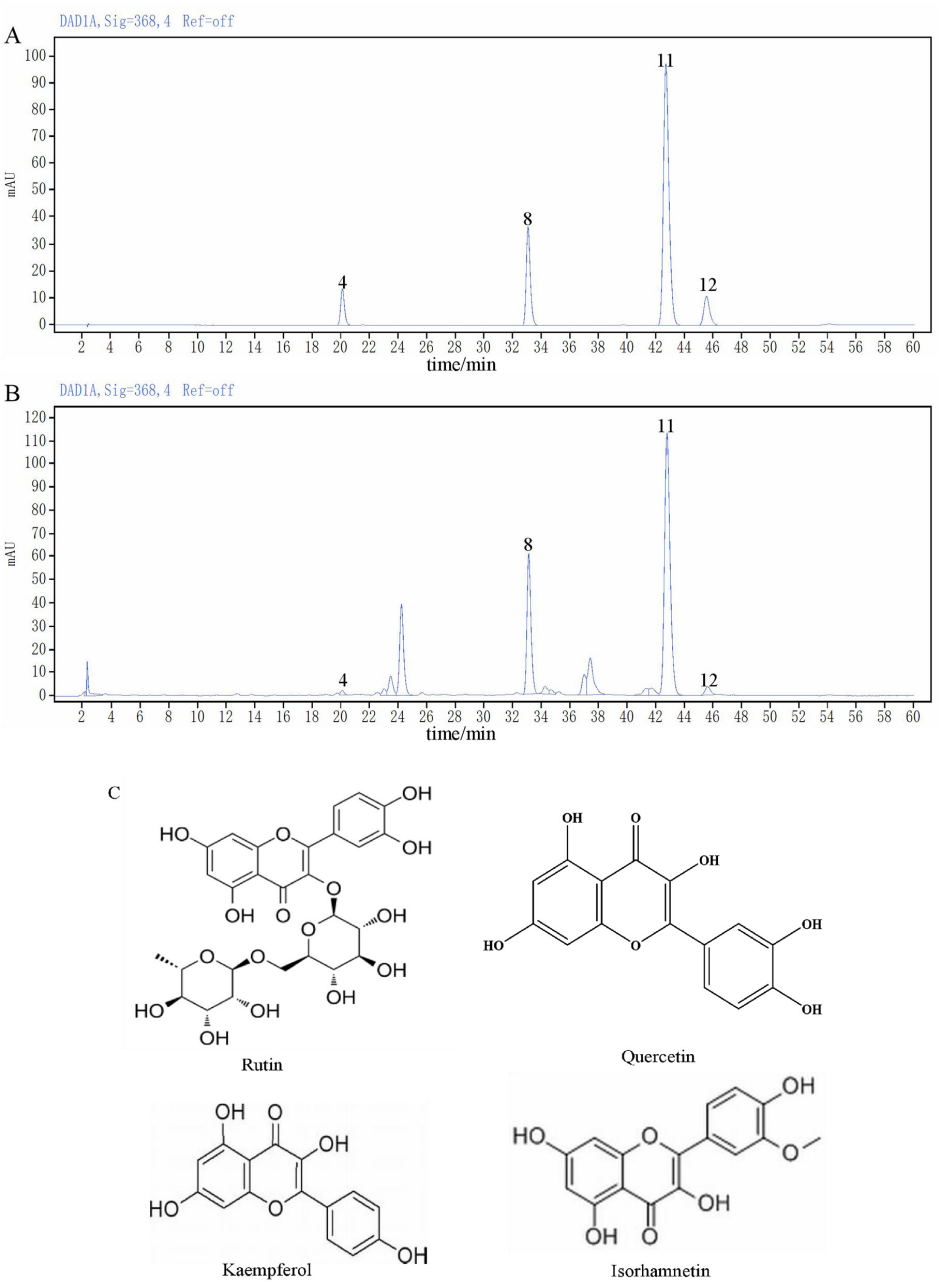
The chromatogram of the mixed reference substance **(A)**, the chromatogram of sample **(B)** and Chemical structures of *four* components **(C)**.

The content of rape bee pollen maca chewable tablets was analyzed using HPLC method. Quantitative analysis was performed on chromatographic peaks 4 (rutin), 8 (quercetin), 11 (kaempferol), and 12 (isorhamnetin) in the fingerprint spectrum. As illustrated in Figure 2B, these four components constitute the primary constituents of the rape bee pollen maca chewable tablets, with quercetin and kaempferol being particularly prominent. HPLC determination was performed on the sample of rape bee pollen maca chewable tablets and the mixed reference solution separately. The results are shown in Figures 2A, B. In addition, Figure 2C shows the structural formulas of each compound. Simultaneously settle the content of each component, as shown in Table 2.

**Table 2:** Determination results of component content of sample.

**Component**	**Content (μg/g)**
Rutin	15.51 ± 5.69
Quercetin	152.85 ± 3.95
Kaempferol	564.98 ± 1.07
Isorhamnetin	11.73 ± 4.76

### 3.3 Effect of chewable tablets containing rape bee pollen and maca in rats with BPH

**Figure 3:**
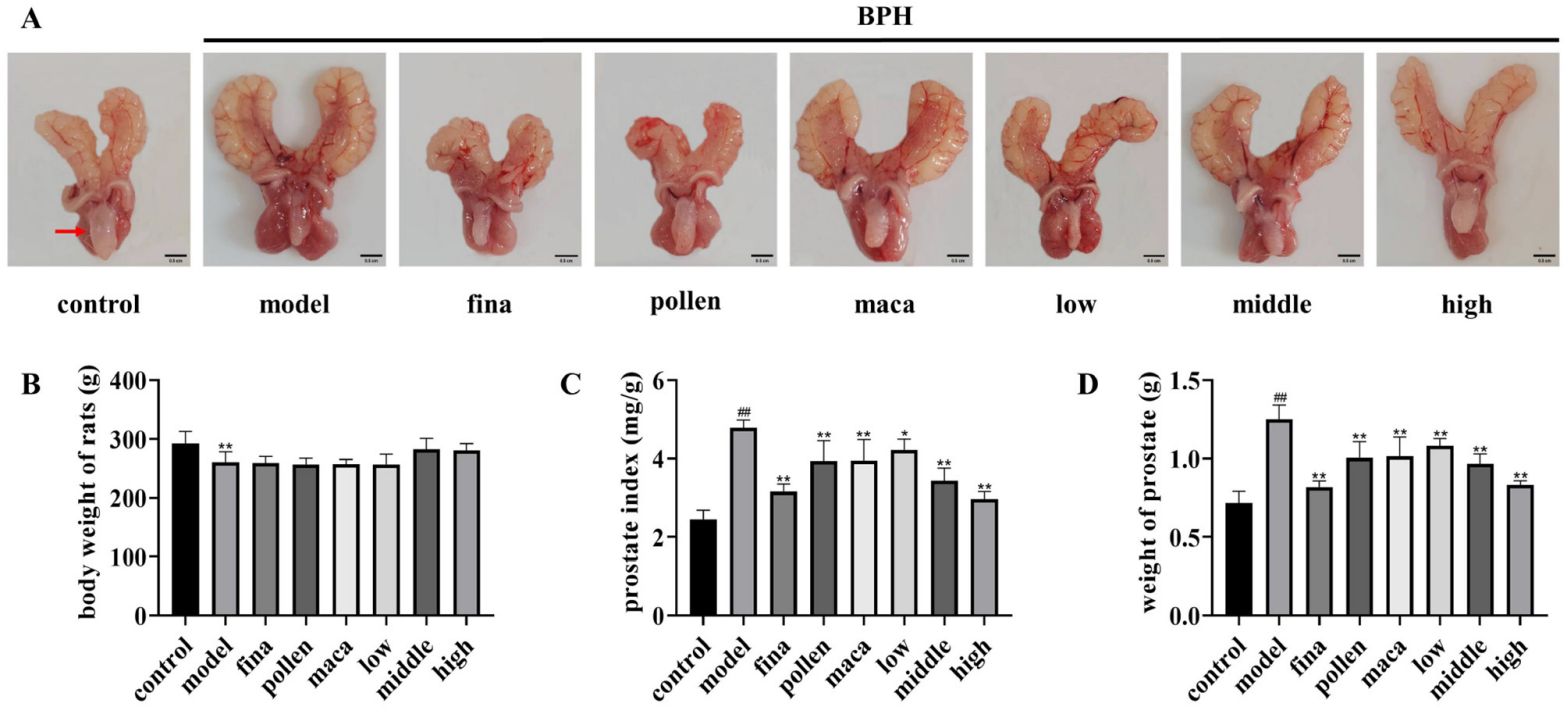
**(A)** Representative images of the prostate from each group. Scale bar = 0.5 cm. (Red arrows indicate rat prostate tissue.) **(B)** Body weight. **(C)** Prostate weight. **(D)** Prostate weight (PW) to body weight (BW) ratio. Data are presented as mean ± standard deviation. ^*##*^*p* < 0.01 vs. rats that did not receive TP injection, *p < 0.05, **p < 0.01 vs. rats injected with TP. Low, middle, and high in the figure represent the low, middle, and high the rape pollen maca chewable tablets groups, respectively.

To confirm whether rape bee pollen maca chewable tablets decreased BPH, a rat model of BPH was induced by testosterone propionate. Prostate tissues from the control group were light pink in color; they had intact morphology, did not adhere to the surrounding tissues, and had no nodules (Figure 3A). However, prostate tissues from the model group were dark red, had a nonsmooth surface, and showed obvious nodules. Tissues from the finasteride, pollen, and maca groups were lighter red in color and showed no obvious nodules. Tissues from the low-dose group of chewable tablets appeared dark red and showed obvious hyperplastic nodules, whereas tissues from the middle- and high-dose groups from chewable tablets were lighter red in color, had a smooth surface, and showed no obvious nodules. The body weight of rats in the model group was significantly lower (*p* < 0.01) than that of rats in the control group. Intriguingly, we observed that rats in the middle- and high-dose groups of the rape pollen maca chewable tablets exhibited greater body weight compared to those in the rape bee pollen and maca groups, though the difference did not reach statistical significance (Figure 3B). The model group showed a significant increase (*p* < 0.01) in both prostate index and prostate wet weight compared with the control group. The prostate index and prostate wet weight in the finasteride group (*p* < 0.01), pollen group (*p* < 0.01), maca group (*p* < 0.01), and the administration of varying doses of chewable tablets group (*p* < 0.01) were significantly lower than those in the model group (Figures 3C, D). Furthermore, our findings revealed that the middle- and high-dose groups of the rape bee pollen maca chewable tablets demonstrated superior efficacy compared to the rape bee pollen and maca treatment groups (Figures 3C, D).

### 3.4 Effect of chewable tablets containing rape bee pollen and maca on serum sex hormone levels in rats

**Figure 4:**
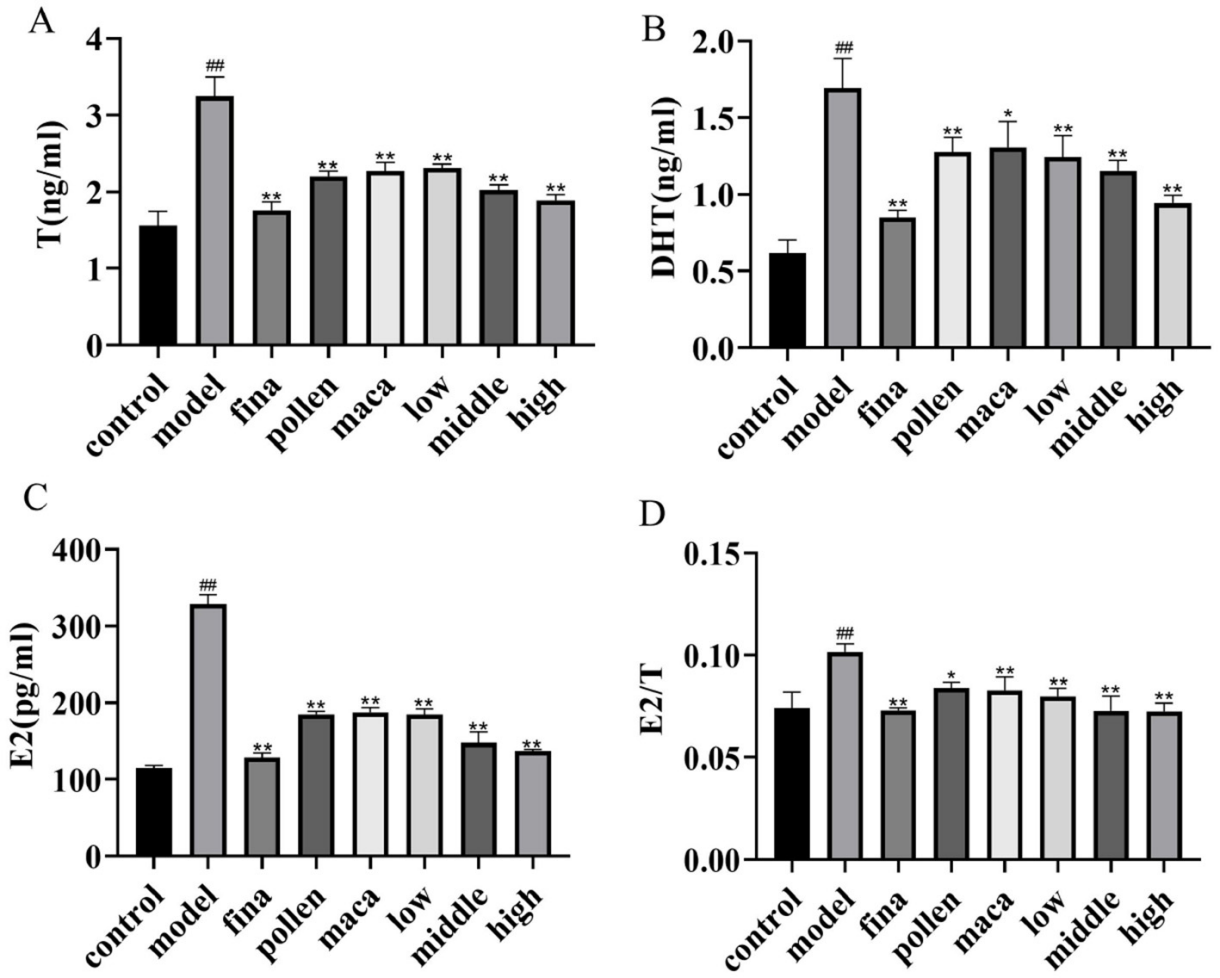
**(A–C)** Serum T, DHT, and E2 levels in rats determined using ELISA. **(D)** The serum E2/T ratio. Data are presented as mean ± standard deviation. ^##^*p* < 0.01 vs. rats that did not receive TP injection, *p < 0.05, **p < 0.01 vs. rats injected with TP. Low, middle, and high in the figure represent the low, middle, and high chewable tablets groups, respectively.

Using ELISA, we looked at the variations in serum hormone levels in each group of rats. As demonstrated in Figures 4A–D, serum levels of T, DHT, and E2 were significantly elevated in the model group compared to the control group, along with an increased E2/T ratio (*p* < 0.01). After treatment with different doses of chewable tablets, the levels of T, DHT, and E2 significantly decreased (*p* < 0.01), as well as the serum E2/T ratio (*p* < 0.01), but did not fully recover to normal levels (vs control). The collective data indicate that chewable tablets containing rape bee pollen and maca can reduce serum hormone levels in rats with BPH, and the chewable tablet is more efficacious than the use of rape bee pollen and maca as standalone agents.

### 3.5 Effect of chewable tablets containing rape bee pollen and maca on serum oxidative stress levels in rats

**Figure 5:**
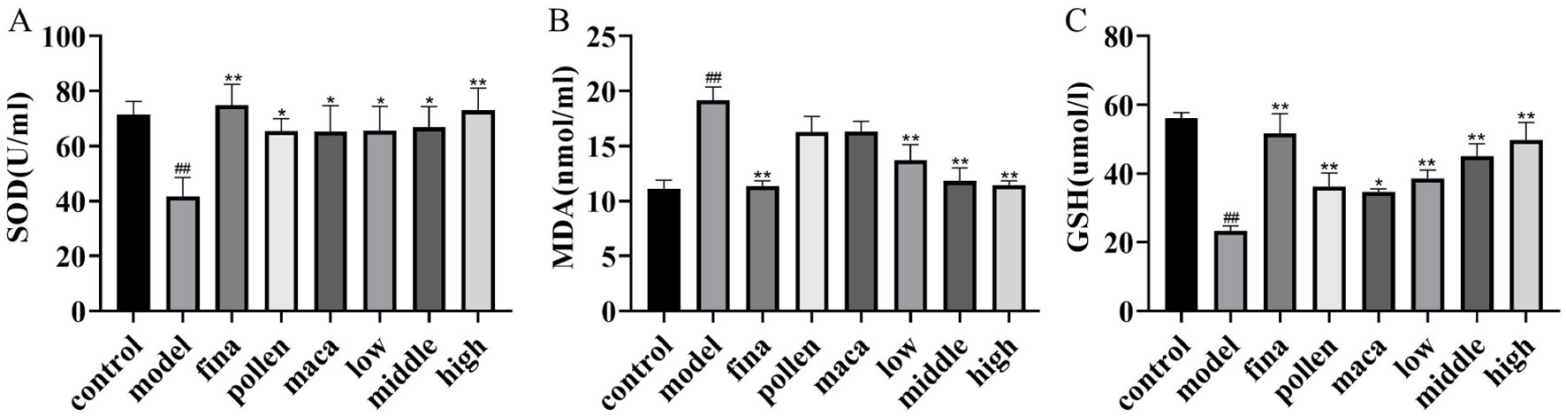
**(A–C)** Serum SOD, MDA and GSH levels in rats determined using Kit. Data are presented as mean ± standard deviation. ^##^*p* < 0.01 vs. rats that did not receive TP injection, *p < 0.05, **p < 0.01 vs. rats injected with TP. Low, middle, and high in the figure represent the low, middle, and high chewable tablets groups, respectively.

Using the kit, we observed alterations in serum oxidative stress levels across all groups of rats. As shown in Figures 5A–C, compared with the control group, the model group rats exhibited significantly decreased serum levels of SOD and GSH along with markedly elevated MDA levels (*p* < 0.01), demonstrating the occurrence of severe redox imbalance in these experimental animals. After treatment with different doses of chewable tablets, the levels of SOD and GSH significantly increased (*p* < 0.01), Meanwhile, the MDA levels were significantly reduced (*p* < 0.01). Furthermore, we found that the combined application of rape pollen and maca demonstrated superior efficacy compared to the individual use of either rape bee pollen or maca alone.

### 3.6 Effect of chewable tablets containing rape bee pollen and maca on the histopathology and serum inflammatory factor levels in rats

**Figure 6:**
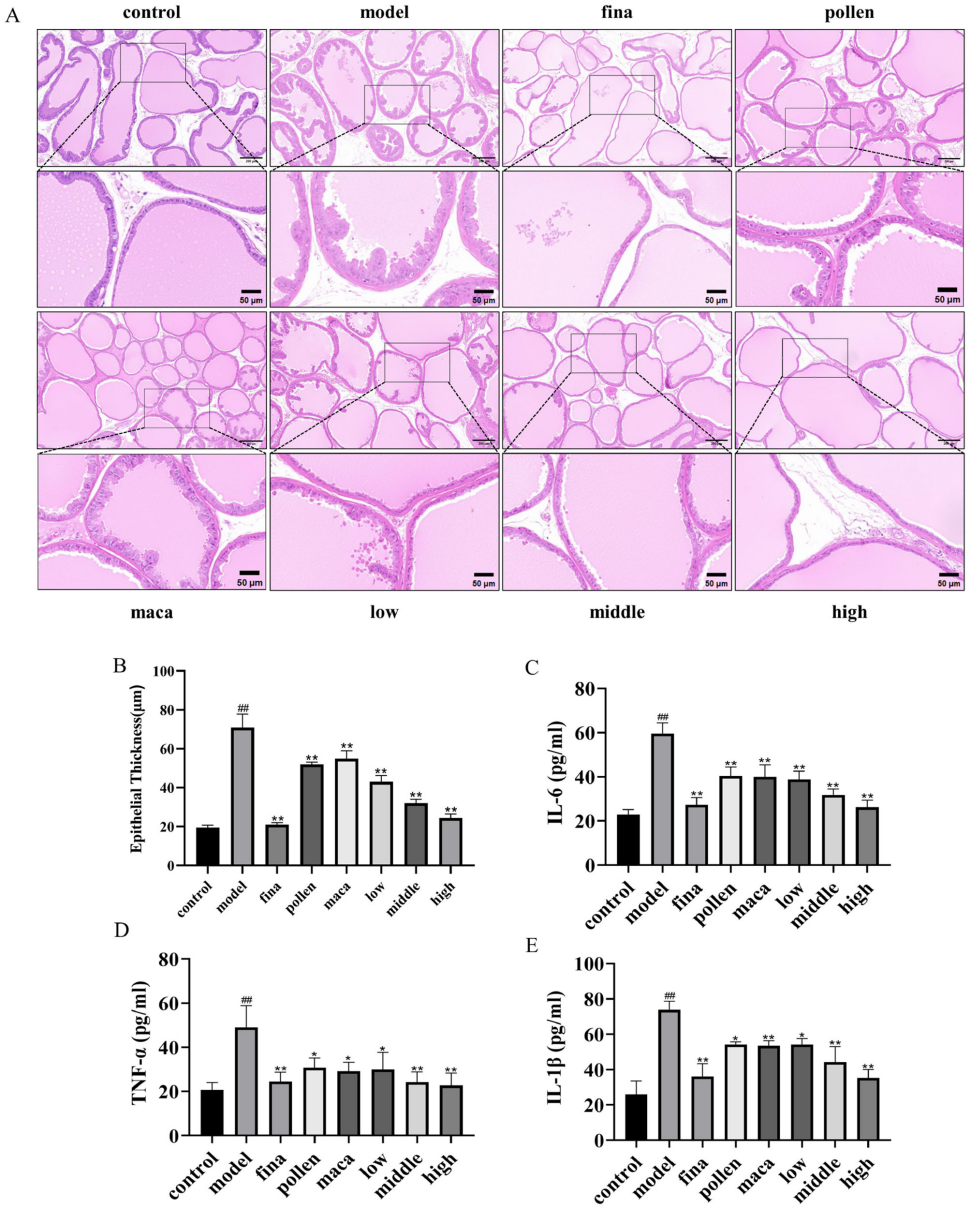
**(A)** H&E staining scale bar = 200 μm. Scale bar = 50 μm. **(B)** Quantification of epithelial thickness. **(C–E)** Serum IL-6, TNF-α, and IL-1β levels in rats determined using ELISA. Data are presented as mean ± standard deviation. ^##^*p* < 0.01 vs. rats that did not receive TP injection; *p < 0.05, **p < 0.01 vs. rats injected with TP. Low, middle, and high in the figure represent the low, middle, and high chewable tablets groups, respectively.

Figure 6A reflects the histopathologic changes in prostate tissues stained with hematoxylin & eosin (H&E). Prostate tissues were distinctly ordered, had a smooth surface, and exhibited fewer acinar epithelial folds, and the epithelial cells were firmly packed in a single column in the control group. In contrast, prostate tissues from model rats exhibited considerable hyperplasia compared with that from control rats. Furthermore, the epithelial cells displayed a multilayer arrangement with more rough folds and increased epithelial cell thickness (*p* < 0.01). In addition, rats in the pollen group, maca group, and low-dose group of chewable tablets showed a significant reduction in prostate epithelial cell thickness (*p* < 0.01) and moderate hyperplasia of the prostate gland, whereas rats in the middle-dose group showed mild hyperplasia of the prostate gland and a substantial reduction in epithelial cell thickness (*p* < 0.01) compared with those in rats in the model group. On the other hand, rats in the high-dose group and finasteride group showed normalization of the prostate gland structure and a significant reduction in epithelial cell thickness (*p* < 0.01) (Figure 6B). Enzyme-linked immunosorbent assay (ELISA) was used to determine variations in serum inflammatory factor levels in each group of rats. The chewable tablet significantly decreased IL-6, TNF-α, and IL-1β levels in the blood (*p* < 0.01) (Figures 6C–E). The collective data indicate that chewable tablets containing rape bee pollen and maca could impede the progression of BPH and indicated chewable tablets to be more efficacious than rape bee pollen or maca used singly.

### 3.7 Effect of chewable tablets containing rape bee pollen and maca on the diversity of gut microbiota of rats

**Figure 7:**
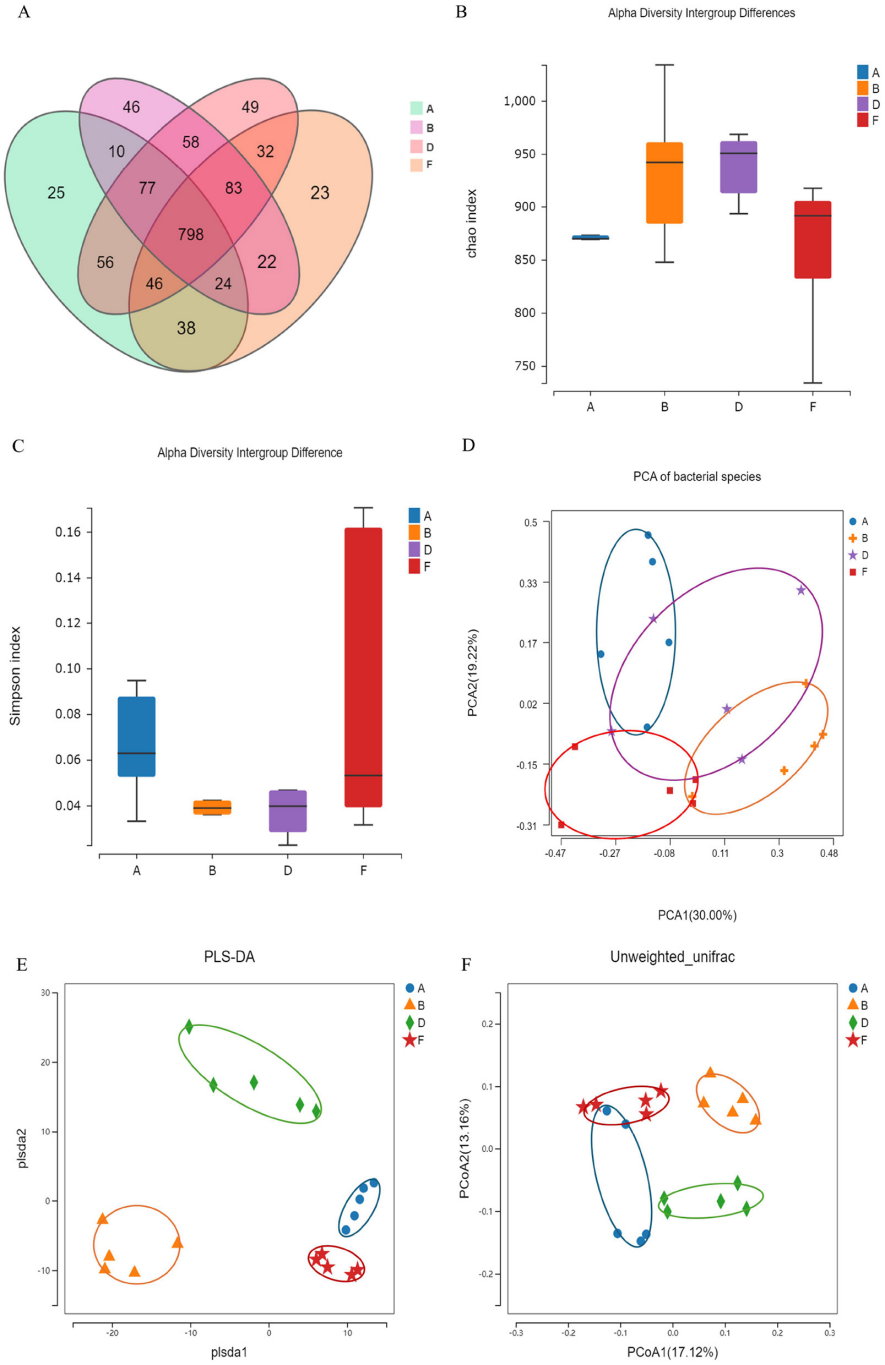
**(A)** Overview of OTUs in diverse groups. **(B)** Chao index. **(C)** Simpson index. Differences were assessed by the Kruskal-Wallis test. **(D)** Principal components analysis (PCA). **(E)** Partial least squares discrimination analysis (PLS-DA). **(F)** Principal Coordinates Analysis (PCoA). **(A, B, D, F)** In the figure represent the control, model, middle-dose chewable tablet, and finasteride groups, respectively.

To investigate the effects of rape pollen maca chewable tablets on the gut microbiota in rats with BPH, we selected the following groups as study subjects: the control group, model group, finasteride group, and middle-dose rape bee pollen maca chewable tablet group. Operational taxonomic unit (OTU) data revealed differences in the gut microbiota among the four groups. A total of 941 OTUs were identified by grouping the pristine reads using a 97% threshold as shown in the Venn diagram (Figure 7A). The model, middle-dose chewable tablet group, finasteride, and control groups shared 798 OTUs. A total of 25 unique OTUs in the control group, 46 in the model group, 49 in the middle-dose chewable tablet group, and 23 in the finasteride group were identified. The Chao and Simpson indices, representing the gut microbiota alpha diversity, are commonly used to assess species richness and evenness. The data revealed that the differences in the Chao index among the groups were not statistically significant (*p* > 0.05). Nevertheless, the Chao index demonstrated an increase in the model and treatment groups compared with the control group, implying a higher abundance of intestinal flora compared with that in the control group (Figure 7B). A comparison of the Simpson index of the intestinal flora in the model group with that of the control group revealed a decrease in the former (*p* > 0.05) (Figure 7C). The dissimilarity in microbial communities between samples or populations is referred to as beta diversity. Principal component analysis and partial least squares discriminant analysis revealed variations in gut microbiota between the model group and other groups (Figures 7D, E). Principal coordinates analysis derived from unweighted Unifrac distances revealed that the model group grouped independently, whereas the other groups exhibited partial cross-aggregation. These findings demonstrated that the intestinal flora composition of rats in the model group differed from that of control rats. Furthermore, treatment with finasteride or the middle-dose chewable tablet led to changes in the intestinal flora composition in rats with BPH (Figure 7F).

### 3.8 Effect of chewable tablets containing rape bee pollen and maca on the composition of gut microbiota

**Figure 8:**
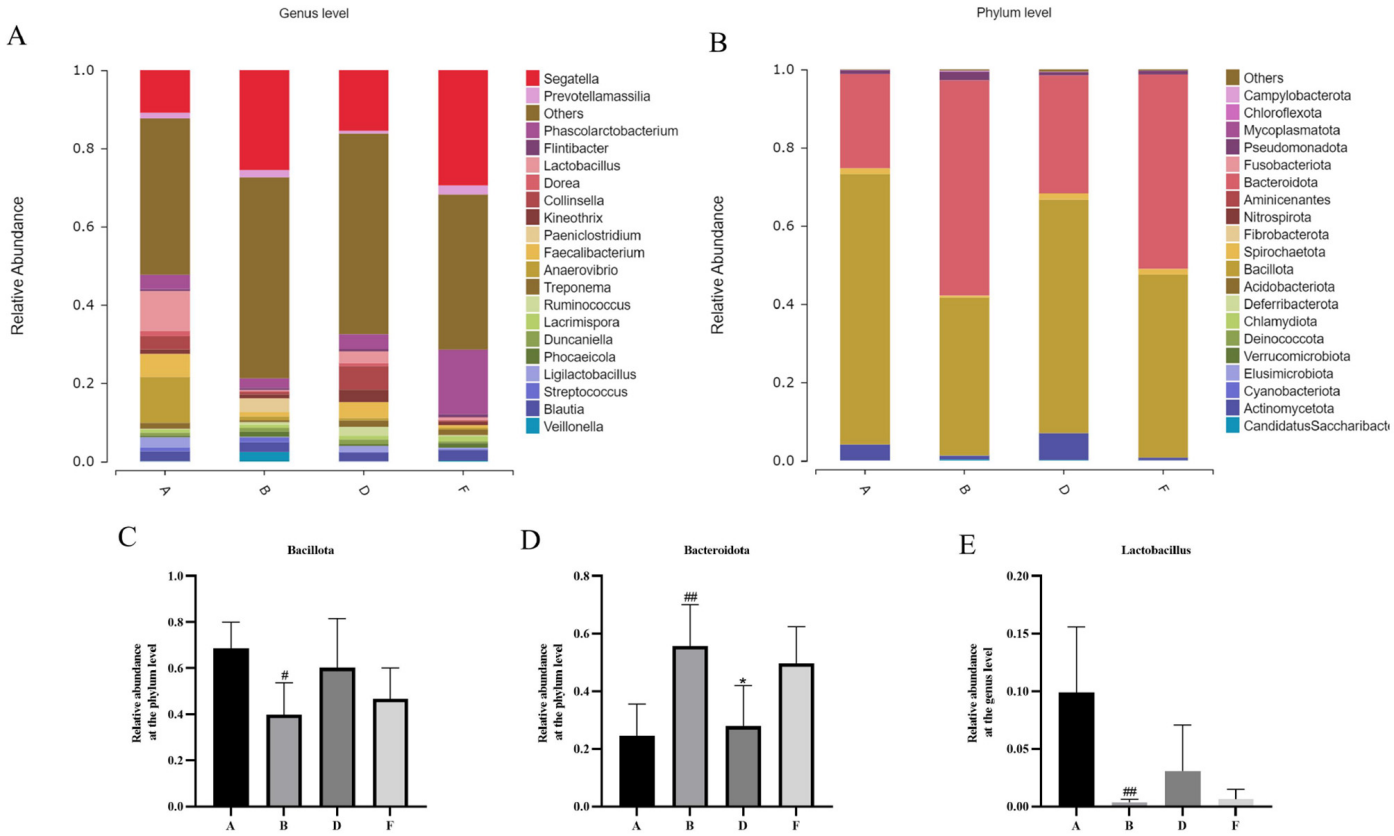
**(A)** Histogram showing the relative abundance of gut bacteria at the phylum level. **(B)** The bar chart shows the relative abundance of intestinal microbes at the genus level. **(C)** relative abundance of bacillota at the phylum level. **(D)** relative abundance of bacteroidota at the phylum level. **(E)** relative abundance of lactobacillus at the genus level. **(A, B, D, F)** in the figure represent the control, model, middle-dose chewable tablet, and finasteride groups, respectively.

Species difference maps were used to show the constitution of the main gut microbiota in different intervention groups; identify significant changes in the gut microbe at the phylum, and genus levels; analyze the reasons for the differences in the diversity of the gut microbiota in different groups; and explain changes in the structure of the intestinal microbiota. A significant increase in *Bacteroidia* (*p* < 0.01) and a significant decrease in *Bacillus* (*p* < 0.05) were noted at the phylum level in the model group compared with that in the control group (Figures 8A, C, D). At the genus level, significant increases were observed in the abundance of *Segatella* and *Prevotellamassilia*, but substantial decreases in the abundance of *Lactobacillus* (*p* < 0.01), *Phascolarctobacterium, Kineothrix, Collinsella*, and *Faecalibacterium* were reported in the model group relative to the control group (Figures 8B, E). The middle-dose chewable tablet group showed a reversal in this trend at various taxonomic levels compared with that in the model group.

### 3.9 Effect of chewable tablets containing rape bee pollen and maca on the IL-6/JAK2/STAT3 signaling pathway

**Figure 9:**
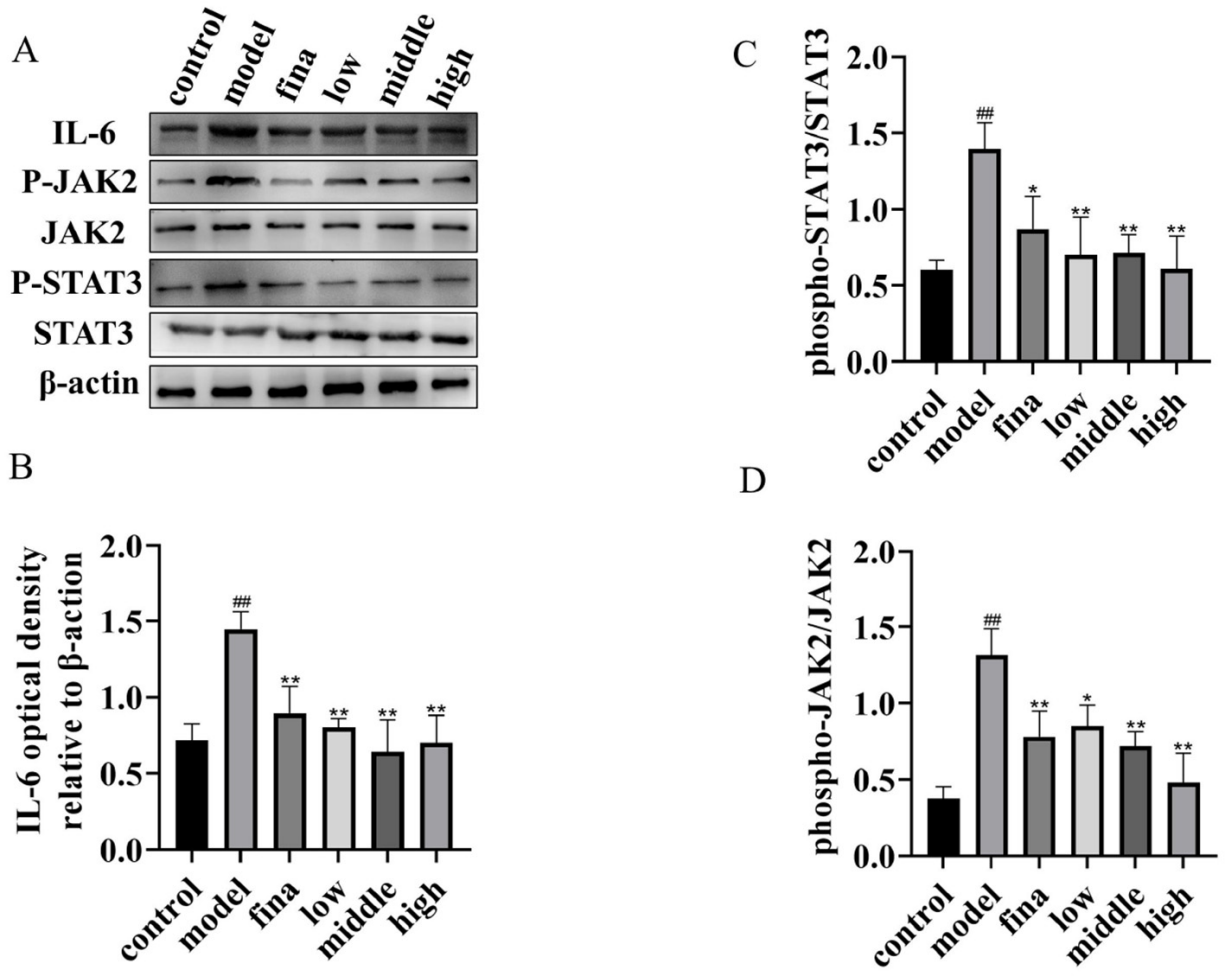
**(A)** Total protein and phosphorylated protein of STAT3 and JAK2, IL-6 proteins. One representative blot was selected from 3 independent experiments. **(B–D)** The relative band intensity was normalized to the relative actin intensity compared with the control. ^##^*p* < 0.01 compared with rats not injected with TP; *p < 0.05, **p < 0.01 compared with the model group.

To further investigate the mechanism of action of rape bee pollen and maca chewable tablets on BPH, we employed Western blotting to detect the expression levels of key proteins in the IL-6/JAK2/STAT3 signaling pathway within prostatic tissues. As shown in Figure 9, compared with the control group, the protein expression levels of IL-6, p-JAK2, and p-STAT3 in the prostatic tissues of model group rats were significantly increased (*p* < 0.01). After drug administration, a marked decrease in the expression levels of IL-6, p-JAK2, and p-STAT3 was observed in the prostate tissues of the treated groups compared to the model group (*p* < 0.01). Our findings indicate that the therapeutic mechanism of rape pollen maca chewable tablets against BPH involves the regulation of the IL-6/JAK2/STAT3 signaling pathway.

## 4 Discussion

This study established a fingerprint of rape bee pollen maca chewable tablets using HPLC, and 12 common peaks were identified as characteristic peaks. Four known components were identified by comparison with the fingerprint of the mixed control solution, Peak 4 was rutin, peak 8 was quercetin, peak 11 was kaempferol, and peak 12 was isorhamnetin. Their amounts were 15.51 ± 5.69 μg/g, 155.85 ± 3.95 μg/g, 564.98 ± 1.07 μg/g, and 11.73 ± 4.76 μg/g, respectively. The fingerprint spectrum of rape bee pollen maca chewable tablets established in this study is simple, stable, and has good repeatability. It can comprehensively reflect the effective chemical components of rape bee pollen maca chewable tablets, which is conducive to the comprehensive analysis of the quality of rape bee pollen maca chewable tablets. Additionally, studies have reported that quercetin and kaempferol exhibit therapeutic effects on benign prostatic hyperplasia induced by testosterone propionate solution (Wang et al., 2021; Fu et al., 2022).

Our findings revealed that the chewable tablet of rape bee pollen and maca showed better efficacy compared with the use of rape bee pollen or maca alone (Figures 3–5). According to clinical medication observations, excessive consumption of rape bee pollen may lead to gastrointestinal reactions such as nausea, vomiting, and loose stools. Additionally, a small number of patients may develop allergic skin reactions, including rash or pruritus (Hemmer et al., 2004). Therefore, the combined application of rape pollen and maca allows for a reduction in the dosage of rape pollen required, thereby mitigating the occurrence of certain adverse reactions. Since both rape pollen and maca serve dual purposes as medicinal agents and food-grade ingredients, they exhibit a low risk of adverse effects and minimal contamination during production processes. Thus, the chewable tablet of rape bee pollen and maca is more widely available than the chemically synthesized drug finasteride. Therefore, this study conducted a pharmacodynamic evaluation of rape pollen maca chewable tablets on benign prostatic hyperplasia, investigated their impact on gut microbiota, and further explored the IL-6/JAK2/STAT3 signaling pathway in prostatic tissues.

It has been established that BPH is influenced by both estrogens and androgens. The prostate's response to estrogens is linked to several processes, such as aromatase expression, apoptosis, and paracrine regulation through prostaglandin E2. E2 to T ratio rises in BPH and malignant prostate tissues due to aberrant expression of aromatase (Wang et al., 2024). 5α reductase (5-AR) converts T, the major circulating androgen in males, to DHT. DHT enters the nucleus by attaching to the AR at the androgen binding site. The DHT/AR complex molecule then attaches to the ARE in the promoter of the target gene, encouraging the growth and survival of prostate epithelial cells (Park et al., 2022; Gong et al., 2023).

In this study, a rat model of testosterone propionate induced BPH was used to determine the efficacy of chewable tablets containing rape bee pollen and maca. Our findings demonstrated that chewable tablets could lead to a notable reduction in prostate volume, epithelial thickness, and serum levels of IL-6, TNF-α, and IL-1β. Inflammation is an important factor in the process of BPH (Cai et al., 2020). Chronic inflammation leads to the overproduction of pro-inflammatory factors, including IL-6, IL-1β, and TNF-α, which induces the overproliferation of prostate epithelial and stromal cells and activates fibrosis to promote signaling and malignant transformation (Cai et al., 2020). We found that chewable tablets group exerted anti-inflammatory activity by significantly inhibiting the IL-6/JAK2/STAT3 signaling pathway. The IL-6/JAK2/STAT3 signaling pathway plays an important role in the development of BPH. IL-6 binds to the IL-6 receptor on the cell membrane and activates intracellular JAK2 and phosphorylating it. Phosphorylated JAK2 further activates STAT3. After dimerization of phosphorylated STAT3, the downstream nuclear transcription factors are activated, which promotes the production and release of IL-6 and exacerbates the inflammatory response (Bowman et al., 2000; Kim et al., 2016; Zhang et al., 2018).

*Lactobacillus* has anti-inflammatory effects, stimulates brain functions, modulates metabolism, and antagonizes intestinal pathogens (Cai et al., 2020). It can metabolize organic acids such as lactic acid, formic acid, acetic acid, and butyric acid, which reduce the intestinal pH and inhibit the growth of acid-intolerant genera in the host intestine (Petrova et al., 2017; Tramontano et al., 2018). *Faecalibacterium* has been demonstrated to confer protection in several studies owing to its anti-inflammatory and symbiotic characteristics. It plays a role in metabolizing acetate into butyrate, which serves as a primary source of energy for colonocytes and functions as an anti-inflammatory agent (Sha et al., 2020; Maioli et al., 2021; Martín et al., 2023). *Collinsella* is responsible for the production of butyric acid and reducing inflammatory diseases (Jeong et al., 2019; Qin et al., 2019). The excessive accumulation of succinic acid in the gut can lead to diarrhea, and *Phascolarctobacterium* can use succinic acid to produce propionic acid (Wu et al., 2017). *Kineothrix* can produce butyrate, which plays a role in modulating inflammation (Choi et al., 2023). *Prevotella* has virulence factors, including adhesins, pili, hemolysins, nucleases, proteases, lipopolysaccharides, exopolysaccharides, and other enzymes, which promote its pathogenicity and survival in the host (Sharma et al., 2022). The overgrowth of *Prevotella* or its translocation across the mucosal barrier enhances the immune response and leads to inflammatory diseases (Iljazovic et al., 2021). The genus *Prevotella* exhibited abnormally elevated abundance in the model group, while the SCFA-producing gut microbiota including *Lactobacillus, Faecalibacterium, Collinsella, Phascolarctobacterium*, and *Kineothrix* were significantly reduced. This imbalance led to an overproduction of pro-inflammatory factors and facilitated the development of BPH. In contrast, chewable tablets group demonstrated an increase in the abundance of beneficial bacteria, including *Faecalibacterium, Collinsella, Phascolarctobacterium, Kineothrix*, and *Lactobacillus*, while concurrently demonstrating a decrease in the abundance of the pro-inflammatory bacterial *Prevotella*. The finasteride group demonstrated an increase in the levels of beneficial bacteria, specifically *Phascolarctobacterium* and *Lactobacillus*, while concurrently demonstrating a decrease in the abundance of the pro-inflammatory bacterial *Prevotella*.

## 5 Conclusion

In conclusion, this study demonstrates that the chewable tablet of rape bee pollen and maca ameliorates testosterone propionate-induced BPH in rats. The protective effects, mediated through the IL-6/JAK2/STAT3 signaling pathway, are associated with reduced inflammation, oxidative stress, and hormonal imbalance, alongside a beneficial modulation of the gut microbiota. In addition, we also established an HPLC fingerprint of rape bee pollen maca chewable tablets. Our findings indicate these chewable tablets containing rape bee pollen and maca as a promising natural therapeutic strategy for BPH management.

## Data Availability

The data presented in the study are deposited in the NCBI Sequence Read Archive (SRA) repository, accession number PRJNA1353094.
